# Comparison of acute intermittent fasting interventions on mood, hunger, sleep quality, and diet satisfaction in females: A crossover experimental design

**DOI:** 10.1371/journal.pone.0356690

**Published:** 2026-08-26

**Authors:** Kristan Amendola, Laura Harris, Rivka Ohayon, Alison K. Shea, Anthea Christoforou, Antony D. Karelis, Jennifer J. Heisz

**Affiliations:** 1 Department of Kinesiology, McMaster University, Hamilton, Ontario, Canada; 2 Department of Agricultural and Environmental Sciences, McGill University, Montreal, Québec, Canada; 3 Department of Neuroscience, Tel Aviv University, Tel Aviv, Israel; 4 Department of Obstetrics and Gynecology, McMaster University, Hamilton, Ontario, Canada; 5 Department of Exercise Science, Université du Québec à Montréal, Montreal, Québec, Canada; Fundación Universitaria del Área Andina, COLOMBIA

## Abstract

The present study utilized a randomized crossover design to compare the short-term effects of time-restricted feeding (TRF) and alternate-day fasting (ADF) on the subjective experiences of young females. Twenty-three females (*M*_age_ = 18.3 ± 0.4 years) participated in this study. Participants were asked to complete three days of TRF and three days of ADF, separated by a four-day washout period. On the day before fasting and on each fasting day, the participants completed an online questionnaire at noon. The questionnaire collected information on mood, hunger, diet satisfaction and subjective sleep quality. Total mood disturbance increased following ADF compared to TRF [condition x time interaction: *F*(3, 57) = 3.35, *p* = .03, η²ₚ = .15], but this effect disappeared by the second fasting day. Hunger increased on fasting days following both TRF and ADF [main effect of time: *F*(3, 57) = 19.82, *p* < .001, η²ₚ = .51, and a condition x time interaction: *F*(3, 57) = 10.08, *p* < .001, η²ₚ = .35]. Diet satisfaction was significantly higher during TRF than ADF [main effect of condition: *F*(1, 19) = 5.21, *p* = .03, η²ₚ = .22]. Neither fasting protocol significantly affected subjective sleep quality. Together, these findings suggest that TRF may offer a more tolerable fasting strategy than ADF, particularly in terms of mood and diet satisfaction, without adverse effects on subjective sleep quality.

## Introduction

Intermittent fasting (IF) refers to dietary regimens that alternate between periods of eating and fasting, varying in duration and frequency. Two of the most commonly practiced IF protocols are time-restricted feeding (TRF) and alternate-day fasting (ADF). TRF typically involves daily fasting periods of 12–20 hours, with unrestricted eating during the remaining hours; a common variation is the 16:8 method, which allocates 16 hours for fasting and 8 hours for eating [1,2]. In contrast, ADF alternates between fasting days and days of normal eating [1]. These protocols have garnered widespread attention due to their metabolic benefits, such as improvements in blood pressure, lipid profiles, and insulin sensitivity [3,4].

While the physiological outcomes of IF are well documented, considerably less is known about its impacts on subjective experiences such as mood, hunger and diet satisfaction—factors that play a critical role in adherence to lifestyle interventions [5–10]. Understanding these psychological and behavioural responses during the early adoption phase is especially important, as initial impressions can influence long-term sustainability [11].

Although IF has been studied in relation to chronic health outcomes, its short-term psychological effects remain understudied, methodologically heterogeneous, and inconclusive. For example, a recent systematic review of 10 studies examining the mood response to long-term fasting interventions ranging from 4 to 16 weeks found no consistent effect on mood scores across populations or protocols [12]. In contrast, two studies tracking mood fluctuations during short-term interventions in healthy females reported increases in negative mood, as measured by the Profile of Mood States (POMS) [13] and a VAS ranging from zero (“not at all”) to 10 (“extremely”) [14].

Hunger, a primary driver of eating behaviour, has also been insufficiently explored in short-term IF. Longer interventions of 3–8 weeks report mixed results—some reporting increased hunger [15], others reporting no change [16]. By comparison, studies involving fasting durations of 12–24 hours consistently report elevated hunger during fasting periods [14,17,18], though differences in fasting duration complicate comparisons.

Diet satisfaction, central to long-term adherence, has received even less attention in IF research. To date, only one study has directly compared satisfaction across fasting protocols during the early adoption phase, finding TRF to be rated more acceptable than ADF or early TRF (i.e., a more restrictive version of TRF with an early eating window), with 71% of participants identifying TRF as the easiest to follow [19]. Moreover, diet satisfaction with TRF also seems to remain stable throughout a 4-week intervention [20].

Subjective experiences such as mood, hunger and diet satisfaction strongly influence behavioural adherence. Lower mood has been associated with reduced exercise adherence [5], heightened hunger predicts poorer dietary compliance [9], and greater diet satisfaction is associated with sustained weight-loss [21]. Sleep may also indirectly influence adherence through its effects on mood [22] and hunger regulation [23], though findings are mixed: some studies report no effect of IF on sleep characteristics [24–28], while others report sleep disturbances [29,30]. Despite the relevance of these variables, little is known about how they fluctuate daily in response to IF during the initial adoption phase.

To date, no study has directly compared TRF and ADF with respect to their short-term effects on mood, hunger, diet satisfaction, and subjective sleep quality within the same individuals. Such a within-subject comparison is essential for identifying which IF protocol is easier to follow during early adoption and may therefore be more sustainable over time. The present study aimed to address this gap by comparing the short-term effects of TRF and ADF on females’ subjective experience of mood, hunger, diet satisfaction, and sleep quality.

## Materials and methods

### Participants

Twenty-three healthy biologically female adults (*M*_age_ = 18.3 ± 0.4 years, range = 18–19 years) participated in this research study. Given the higher prevalence of young adult Canadian females practicing IF compared to males [31], biologically female adults were chosen as the target population to increase the relevance and applicability of the study results. Participants were recruited using advertisement via emails, social media, McMaster Sona Systems and posters on the McMaster campus and surrounding areas. An a priori power analysis for a within-subjects repeated measures ANOVA was conducted using G*Power software. Assuming a moderate effect size of 0.25 based on previous fasting research [32], with 80% power, and an alpha level of .05, it was estimated that 16 participants were needed. To account for a 20% dropout rate, we aimed to recruit at least 20 participants. We overrecruited, resulting in a final sample size of 23.

Participants were included if they were English-speaking females between the ages of 18–30 years old, with a body mass index (BMI) between 18.5–24.9 kg/m^2^, physically active (≥ 150 minutes of moderate-vigorous intensity physical activity per week), having a normal menstrual cycle for at least three months, non-smokers, and consuming no more than one alcoholic beverage per day. Participants were excluded if they were: self-reported pregnant or breastfeeding, diagnosed with a severe chronic disease (cancer, diabetes, cardiovascular, psychiatric—bipolar disorder, major depressive disorder, or schizophrenia), diagnosed with an eating disorder, engaged in a recent fasting practice (intentionally fasting for more than 16 hours per day in the last two weeks), used antidepressant medication, or had substantial weight lost (more than 5% of their body weight) in the last three months.

Participants were screened for eligibility and provided written informed consent. The recruitment period took place between January and March of 2024. This study was approved by the Hamilton Integrated Research Ethics Board (HiREB).

### Study design and procedure

The study employed a crossover experimental design to compare changes in mood, hunger and diet satisfaction over time between two popular fasting protocols. Eligible participants were randomized using a random number generator to complete both the TRF and ADF protocols in counterbalanced order. During the initial in-lab session, participants’ height, weight, mental health status, and demographic information were collected. All participants began their intervention the day after confirming the start of their menstrual cycle (follicular phase) to control for hormonal fluctuations that may impact mood [33]. However, we did not assess hormonal differences within the follicular phase and, therefore, cannot confirm that participants were in equivalent hormonal states during the two intervention periods. It should be noted that protocol one occurred approximately on cycle days 2–5, while protocol two occurred approximately on cycle days 10–13. Each fasting protocol lasted four days, with a four-day washout period between protocols to minimize carryover effects from the previous fast. This washout duration exceeds that of previous fasting studies, which have used a 24-hour washout period [17,34]. A four-day protocol length was chosen to ensure that the total fasting duration was consistent across protocols (i.e., 48 hours), whether this be in the form of three 16-hour TRF fasts or two 24-hour ADF fasts.

Each day of both fasting protocols, participants were asked to complete an online survey at 12:00 PM. Day 1 provided a baseline and was collected before beginning each fasting protocol. For the TRF protocol, participants were instructed to fast for 16 hours each day from 8:00 PM until 12:00 PM the following day; therefore, for TRF days 2, 3 and 4, the online survey was completed at the end of a 16-hour fast. For the ADF protocol, participants were instructed to alternate between a 24-hour fast, which took place from 12:00 PM to 12:00 PM the next day, and a day of regular eating; therefore, for ADF days 2 and 4, the online survey was completed at the end of a 24-hour fast, while for ADF day 3, the online survey was completed after a day of regular eating. These specific times were chosen to control for potential time-of-day effects that may influence mood [35]. Participants were instructed to break their fast only after submitting the online survey.

Upon completion of the study, participants received a $50 Visa gift card, either alone or in addition to two credits on McMaster University's SONA system. Those who dropped out received partial compensation based on the duration of their participation.

### In-lab baseline measures

#### Anthropometric measures.

Body weight was measured using an electronic scale and standing height (± 0.1 cm) was determined using a wall stadiometer (Oregon Rule Co., Oregon, United States). BMI was then calculated by dividing weight in kilograms by height in meters squared.

#### Mental health.

As part of the sample characterization, baseline mental health was assessed using the Depression, Anxiety and Stress Scale (DASS-21). The DASS-21 is a short-form version of the original 42-item validated questionnaire [36,37]. It evaluates symptoms related to anxiety, depression and stress using a 4-point Likert scale for each item, and a total score ranging from 0 to 63, with higher scores representing greater symptom severity [36] and normal scores for depression, 0–9; anxiety, 0–7; and stress, 0–14 [36,38].

#### Disordered eating.

Disordered eating symptomatology was also assessed at baseline using the Eating Disorder Examination Questionnaire 6.0 (EDE-Q 6.0). The EDE-Q 6.0 includes 28 questions to assess symptoms of disordered eating [39]. Questions 1–12 and 19–28 are assessed using a 6-point Likert scale from 0 (indicating symptoms on no days) to 6 (indicating symptoms every day), while questions 13–18 are in an open numeric format [39]. It provides subscale scores for restraint, eating concern, shape concern, and weight concern, as well as an overall ‘global’ score [39]. Subscale scores are calculated by adding relevant items for each subscale and dividing by the total number of items within the subscale [39]. A global score is calculated by adding the subscale scores together and dividing by the four subscales [39]. A global score of ≥ 2.8 for females indicates clinically significant eating disorder symptoms [40].

### Daily measures

All daily questionnaires were collected via an online survey administered through the LimeSurvey platform.

### Mood

Mood was assessed using the Profile of Mood States Second Edition – Adult (POMS 2A), a 65-item self-report questionnaire that captures current affective states in adults. Participants responded to a list of items describing how they felt “*right now,”* using a Likert scale from 0 (“not at all”) to 4 (“extremely”). POMS 2A was used to calculate total mood disturbance (TMD) reflecting the total sum of negative mood states (anger-hostility, confusion-bewilderment, depression-dejection, fatigue-inertia, and tension-anxiety) minus vigour-activity, with higher scores indicating greater negative mood disturbance [41]. Raw scores were converted to standardized T-scores with a mean of 50 and a standard deviation of 10 [42].

### Hunger

Participants rated their hunger level on a visual analogue scale (VAS) from 1 (“starving, no energy, very weak”) to 10 (“extremely stuffed, nauseous”), with lower scores representing greater hunger using the hunger-satiety scale developed at the University of California, Berkeley (https://uhs.berkeley.edu/sites/default/files/wellness-hungersatietyscale.pdf). All scores were reverse-coded by subtracting the original score from 11 (i.e., the sum of the minimum and maximum scores), so that higher scores indicated greater hunger.

### Diet satisfaction

Diet satisfaction was assessed by asking participants to rate their satisfaction with the diet using a Likert scale from 1 (“I am not enjoying this diet at all”) to 5 (“I am really enjoying this diet”).

### Sleep

Self-reported sleep quality was assessed using a question from the Consensus Sleep Diary [43] where participants rated their sleep quality from the previous night on a Likert scale from 0 (“very poor”) to 4 (“very good”).

### Adherence

Self-reported adherence was assessed using a yes/no response to the following question: “Did you follow the fasting protocol today as directed?” Participants who broke protocol (i.e., the fast) were asked to provide a brief explanation and list any consumed food. As a secondary check, participants were also asked to report the start and end times of their fast.

Physiological markers of adherence were assessed using ketone and glucose levels measured by the participant at home during the same time they completed the daily survey (12:00 PM). We used the FreeStyle Precision Neo portable device (Abbott Diabetes Care Inc, Ontario, Canada), which has been shown to provide accurate and reliable results for measuring blood ketone and glucose levels [44]. Each participant was taught how to use the device and given an instruction sheet to take home. Ketone and glucose values were reported in the online survey along with the time of the reading.

### Adverse events and major life events

Participants were asked to report if they experienced symptoms such as headaches or nausea and asked to describe any other adverse events that we may not have listed. Participants were also asked to report if any major life event had occurred that day (e.g., death of a loved one, breakup, career change, injury, etc.) that could have impacted their mood.

### Statistical analyses

Separate two-way repeated measures ANOVAs were conducted to examine fasting-related changes in mood, hunger, and sleep quality, with within-subjects factors of condition (fasting protocol: TRF, ADF) and time (days 1–4). Diet satisfaction was evaluated using the same model, but with three levels of time (days 2–4). Counterbalance order was included as a covariate in these analyses. To correct for multiple comparisons, the Holm-Bonferroni method was applied to control for family-wise error in the post-hoc analyses [45]. Given normality violations for ketone and glucose data, Friedman tests were used to assess changes in ketone and glucose levels across the four days during each fasting protocol. Two outliers were removed from the glucose data for TRF 1 (76.6 mmol/L) and TRF 2 (87 mmol/L) prior to analysis, as the reported values were physiologically impossible, suggesting a reporting error. Post hoc Spearman’s correlations were conducted to assess the relationships between TMD and ketone and glucose levels on day 2 of TRF and ADF. A McNemar test was used to determine whether there was a significant difference between the number of participants reporting an adverse event following each fasting protocol. Missing data were handled using pairwise deletion, where specific cases were deleted if data were missing, while all available data were included in the analyses.

## Results

### Flow diagram

**Fig 1:**
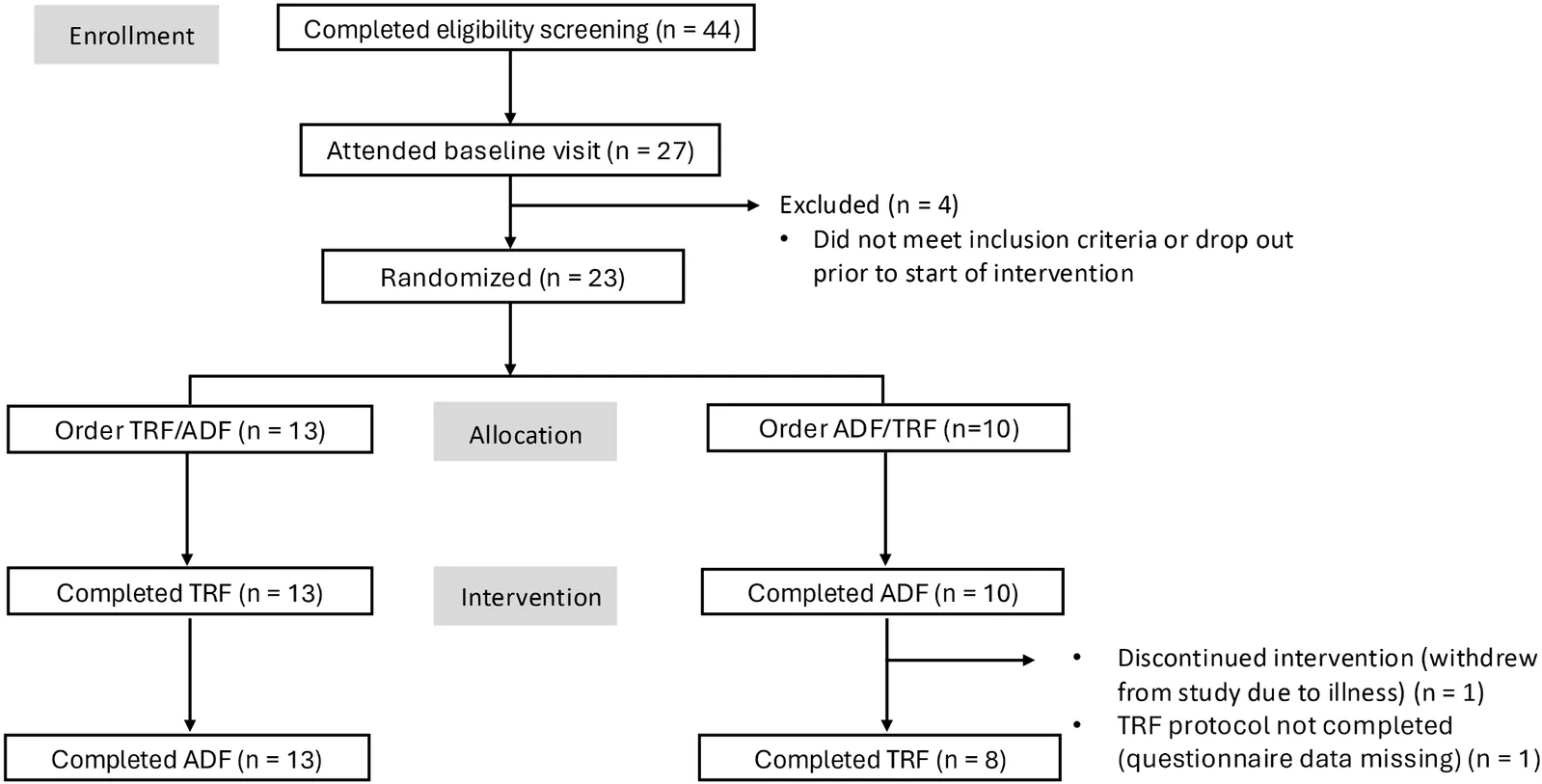
Study flow diagram from enrollment to data analysis.

A flowchart of the study can be found in Fig 1. Forty-four participants completed the eligibility screening questionnaire; however, only 27 participants attended the in-lab session, including three participants who did not meet the BMI inclusion criteria and thus were excluded from the study and one participant who dropped out prior to beginning the intervention. This resulted in a total of 23 participants who were randomly assigned to one of two condition orders using a random number generator. Thirteen participants completed the protocol in order 1 (TRF first, followed by ADF), and 10 individuals completed the protocol in order 2 (ADF first, followed by TRF). One participant dropped out of the study after day 5 due to illness unrelated to the study; however, the data collected during that time were included in our analyses. Another participant completed only the ADF protocol, along with one day of TRF. While their ADF data were analyzed, the TRF data were excluded from analysis because we did not have complete data for the protocol. Therefore, while a total of 22 participants completed the study, only 21 participants completed the full TRF protocol, while 23 participants completed the full ADF protocol.

### Baseline Measures

Table 1 shows the demographic and baseline characteristics of the participants. Participants were on average 18–19 years old (18.3 ± 0.4), with a normal BMI (22.1 ± 1.6 kg/m^2^), recreationally active (249.9 ± 133.5 min/week of moderate to vigorous physical activity), scored “normal” on the DASS-21 with respect to symptoms of depression (7.7 ± 7.2), anxiety (6.9 ± 6.1), and stress (9.6 ± 6.1), and scored “normal” on the EDE-Q 6.0 with respect to disordered eating symptomatology (1.3 ± 0.8). Most participants reported having more than enough income to meet their needs (60.9%), were of East and South Asian descent (39.1% and 30.4%, respectively), and the majority of participants had no history of religious fasting (82.6%).

**Table 1:** Demographic characteristics of participants at baseline.

		*n*	*M*(*SD*)
	Age (years)	23	18.3 (0.4)
	Height (m)	23	1.61 (0.05)
	Weight (kg)	23	58.0 (5.7)
	BMI (kg/m^2^)	23	22.1 (1.6)
	MVPA (min/week)	23	249.9 (133.5)
	DASS-21 Depression Score (/42)	23	7.7 (7.2)
	DASS-21 Anxiety Score (/42)	23	6.9 (6.1)
	DASS-21 Stress Score (/42)	23	9.6 (6.1)
	EDE-Q (/6)	23	1.3 (0.8)
		***n***	***%***
	History of Religious Fasting	4	17.4%
Income	More than enough	14	60.9%
	Just enough	7	30.4%
	Not enough	1	4.3%
	Prefer not to answer	1	4.3%
Race	East Asian	9	39.1%
	South Asian	7	30.4%
	White	4	17.4%
	Latin American	1	4.3%
	Southeast Asian	1	4.3%
	Biracial	1	4.3%
	Black	0	–
	Indigenous	0	–
	Middle eastern	0	–
	Do not know	0	–
	Prefer not to answer	0	–
	Other	0	–

### Daily measures

All daily measures are reported in Table 2.

**Table 2:** Mean (SD) and sample size of mood, hunger, diet satisfaction and sleep by fasting protocol, with paired-samples t*-*test comparisons of baseline values.

	TRF				ADF				*Baseline comparison of TRF Day 1 vs. ADF Day 1*
	*Day 1* *baseline*	*Day 2* *16-h fast*	*Day 3* *16-h fast*	*Day 4* *16-h fast*	*Day 1* *baseline*	*Day 2* *24-h fast*	*Day 3* *No fast*	*Day 4* *24-h fast*	
**Ketones**	0.3 (0.6)	0.9 (1.4)	0.9 (1.8)	0.9 (2.1)	0.3 (0.6)	0.8 (1.3)	0.2 (0.4)	1.3 (2.2)	*t*(19) = -0.3, *p* = .78
***n***	21	20	21	21	22	23	23	22	20
**Glucose**	5.0 (1.1)	3.9 (1.4)	4.2 (1.2)	4.3 (1.2)	5.4 (1.2)	4.0 (0.5)	5.2 (1.0)	4.0 (1.1)	*t*(18) = -0.9, *p* = .37
***n***	20	19	21	20	22	23	23	22	19
**Mood Disturbance**	48.9 (10.9)	50.9 (10.1)	49.1 (13.9)	48.1 (11.2)	47.6 (12.2)	55.0 (12.3)	47.5 (11.9)	52.2 (14.9)	*t*(20) = 0.1, *p* = .90
***n***	21	21	21	21	23	23	23	23	21
**Hunger**	5.5 (1.2)	6.4 (1.3)	6.9 (1.5)	6.2 (1.4)	5.1 (1.2)	7.6 (1.4)	4.7 (1.2)	7.2 (1.4)	*t*(20) = 1.1, *p* = .28
***n***	21	21	21	21	23	23	23	23	21
**Diet Satisfaction**		3.5 (1.1)	3.6 (1.2)	3.6 (1.2)		2.6 (1.0)	3.1 (1.2)	2.7 (1.2)	
***n***		21	21	21		23	23	23	
**Sleep Quality**	2.3 (1.1)	2.4 (1.2)	2.3 (1.1)	2.4 (1.1)	2.0 (1.0)	2.4 (1.1)	2.3 (1.0)	2.1 (1.1)	*t*(20) = 0.9, *p* = .38
***n***	21	21	21	21	23	23	23	23	21

*Note*. The hunger values presented in this table represent the reverse scores of the Hunger-Satiety scale, where higher scores indicate more severe hunger.

### Manipulation checks

Self-reported adherence was 100% during the TRF protocol and 98% during the ADF protocol. Two participants reported minor deviations from the ADF regimen (i.e., had milk with their tea).

Ketone and glucose levels served as physiological markers of adherence. For ketones, there was a significant main effect of time for both the TRF [χ^2^(3) = 15.73, *p* = .001, Kendall’s W = .26] and the ADF protocols [χ^2^(3) = 27.15, *p* < .001, Kendall’s W = .41]. Compared with baseline (day 1), ketone levels were significantly higher on days 2, 3, and 4 of TRF. Ketone levels were also significantly higher compared to the baseline fed state (day 1) on days 2 and 4 of ADF.

For glucose, a significant main effect of time was observed for ADF [χ^2^(3) = 31.40, *p* < .001, Kendall’s W = .50], with significant decreases on days 2 and 4 relative to baseline state. No significant effect was observed for TRF [χ^2^(3) = 7.13, *p* = .068].

### Mood disturbance

**Fig 2:**
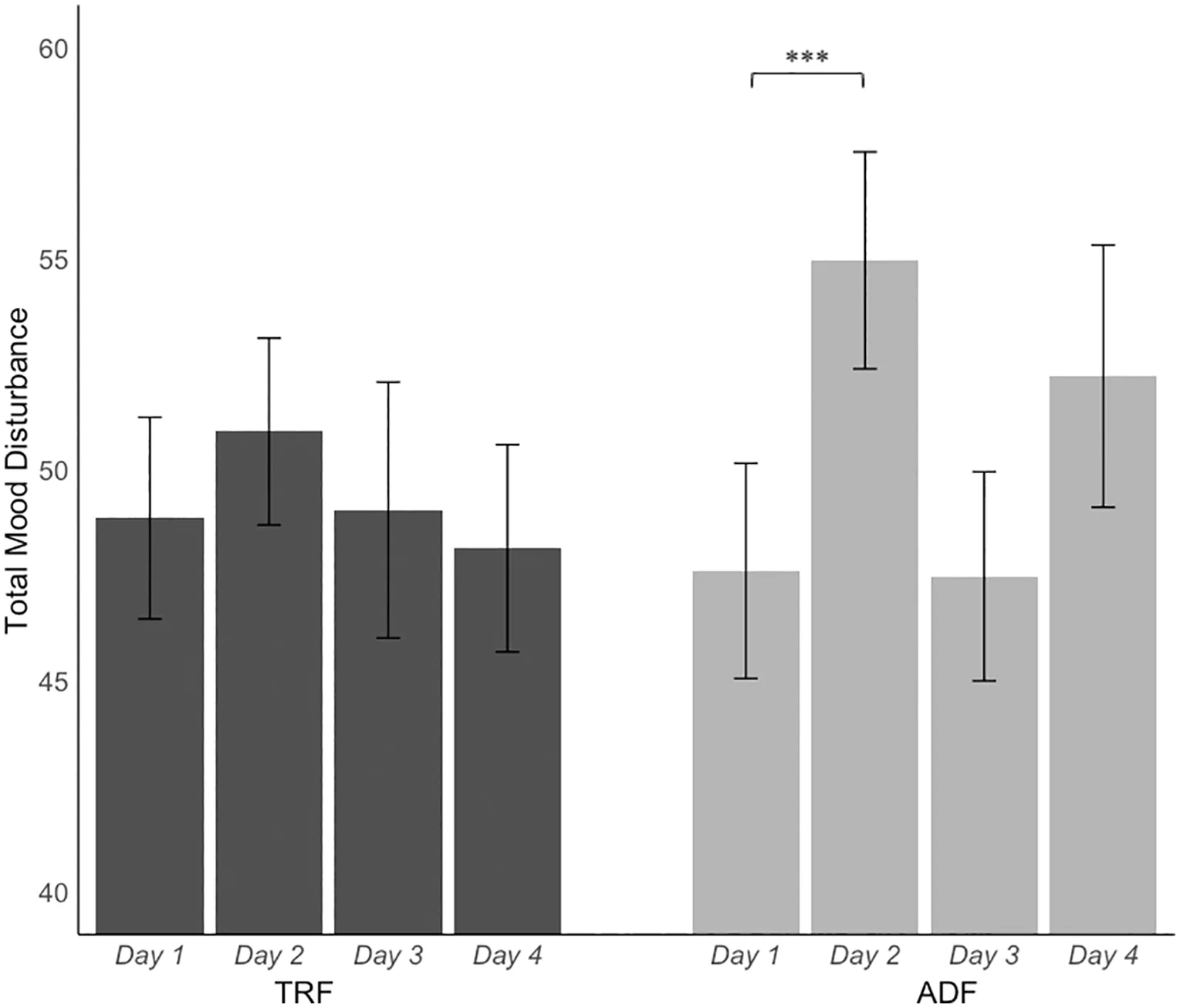
Average total mood disturbance T-scores across fasting protocols. Higher scores indicate greater negative mood disturbance. Error bars represent standard error. Asterisks represent *p*-values of post-hoc tests that remained significant after Holm-Bonferroni correction, where *** = *p* < .001.

A significant condition by time interaction was observed for mood, as rated using the POMS 2A scale [*F*(3, 57) = 3.35, *p* = .03, η²ₚ = .15]. As depicted in Fig 2, a post hoc comparison indicated that mood disturbance increased from day 1 (fed) to day 2 (fasted) during ADF (*p* < .001, Cohen’s *dz* = 0.84), with a large effect size. In the event of a significant interaction, planned post hoc comparisons were conducted between baseline and days 2, 3, and 4 for each protocol. No other significant mood disturbances were observed for ADF relative to day 1, and no significant mood disturbance was observed for TRF relative to day 1. While there was no significant main effect of intervention order for total mood disturbance [*F*(1, 19) = 1.37, *p* = .26, η²ₚ = .07], a significant interaction between intervention order and condition-by-time appeared [*F*(3, 57) = 3.04, *p* = .04, η²ₚ = .14]. A Spearman’s correlation revealed no significant relationship between TMD and ketone levels on day 2 of ADF [*r*_s_(21) = −.01, *p* = .97] or TRF [*r*_s_(18) = −.13, *p* = .59]. Similarly, no relationships were found between TMD and glucose levels on day 2 of ADF [*r*_s_(21) = .28, *p* = .20] or TRF [*r*_s_(17) = .36, *p* = .13].

### Hunger

**Fig 3:**
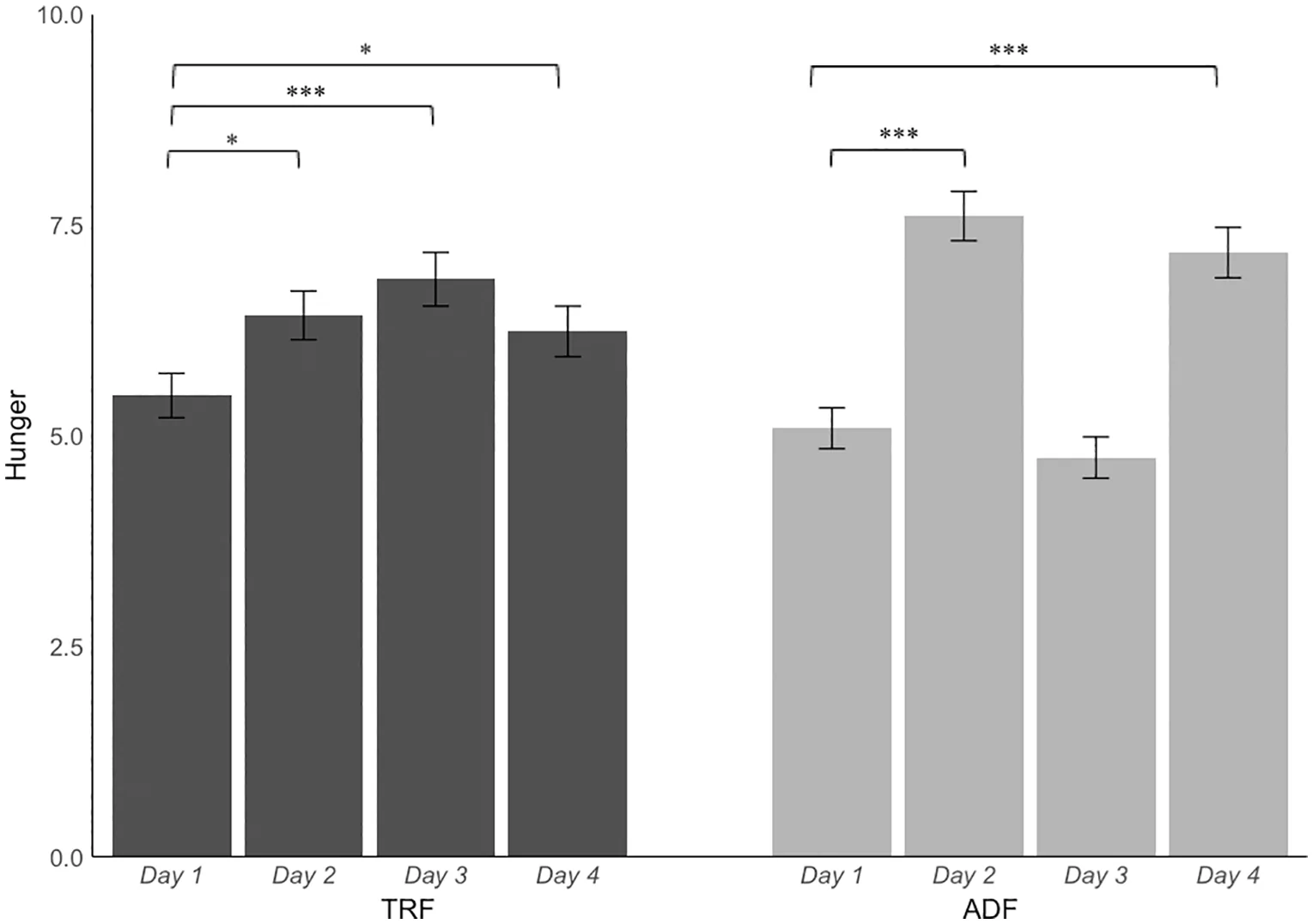
Average hunger scores over time in each fasting condition. Note that the Hunger-Satiety scale was reverse-scored, where higher scores indicate more severe hunger. Error bars represent standard error. Asterisks represent *p*-values of post-hoc tests that remained significant after Holm-Bonferroni correction, where * = *p* < .05, *** = *p* < .001.

There was a main effect of time [*F*(3, 57) = 19.82, *p* < .001, η²ₚ = .51], and a condition by time interaction [*F*(3, 57) = 10.08, *p* < .001, η²ₚ = .35]. As depicted in Fig 3, relative to baseline (day 1), hunger increased (reflecting higher scores due to reverse coding) for TRF on days 2 (*p* = .01, Cohen’s *dz* = 0.62), 3 (*p* < .001, Cohen’s *dz* = 1.08) and 4 (*p* = .02, Cohen’s *dz* = 0.53). Hunger also increased during ADF on days 2 and 4 (*ps* < .001, Cohen’s *dz* values > 0.8). There was no significant main effect of intervention order or interactions between intervention order and condition, time, or condition-by-time for hunger (all *ps* > .05).

### Diet satisfaction

**Fig 4:**
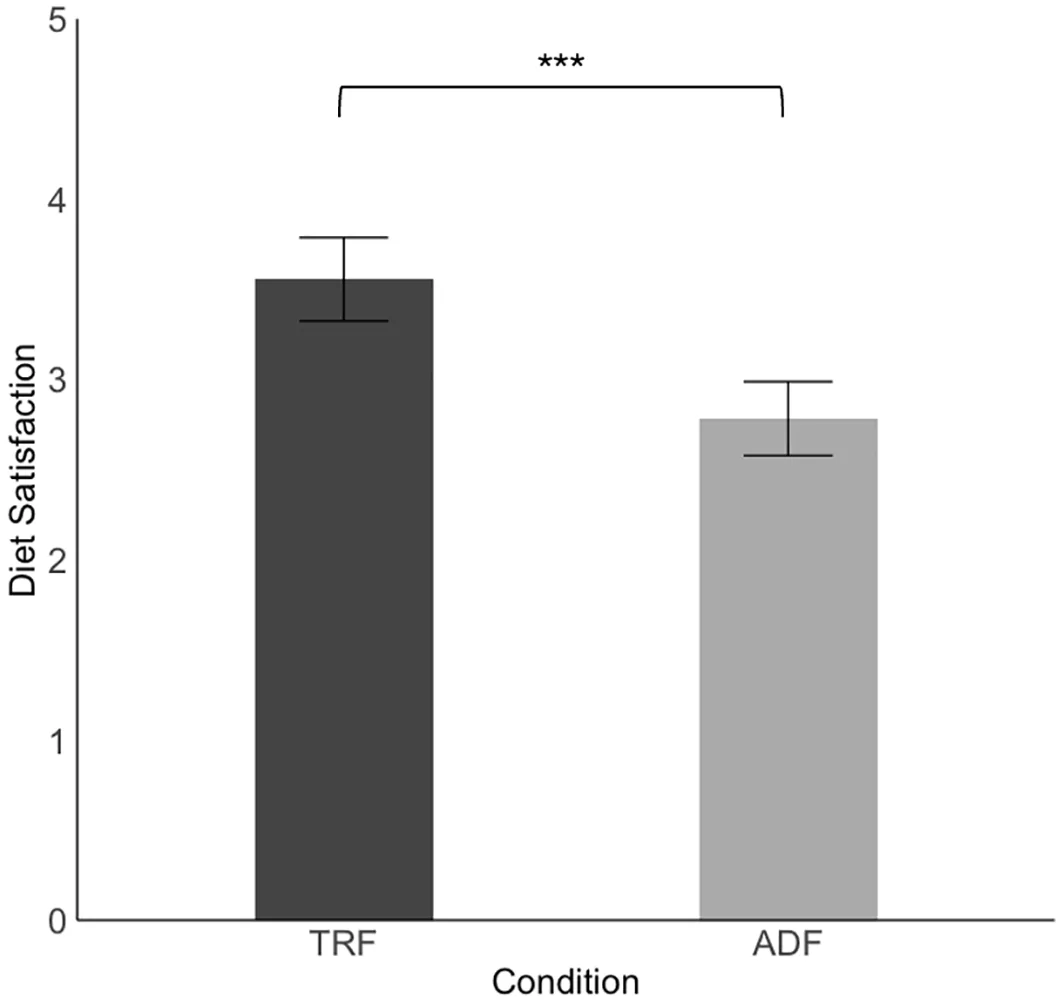
Diet satisfaction was higher for TRF than ADF. Error bars represent standard error. Asterisks represent *p*-values of post-hoc comparisons that remained significant after Holm-Bonferroni correction, where *** = *p* < .001.

A main effect of condition [*F*(1, 19) = 5.21, *p* = .03, η²ₚ = .22] indicated that diet satisfaction was greater for TRF than ADF, as depicted in Fig 4. A main effect of time was also observed [*F*(2, 38) = 4.02, *p* = .04, η²ₚ = .18]; however, post hoc comparisons were not significant. Thus, there was no evidence for a change in diet satisfaction over time within either protocol. While there was no significant main effect of intervention order for diet satisfaction [*F*(1, 19) = 0.20, *p* = .66, η²ₚ = .01], a significant interaction between intervention order and condition appeared [*F*(1, 19) = 5.19, *p* = .03, η²ₚ = .22].

### Sleep quality

Subjective sleep quality was not significantly impacted by the intervention (*ps* > .05).

### Adverse events

Table 3 displays the total number of each adverse event reported across fasting protocols. Numerically, more adverse events were reported for ADF (*M* = 4.70, *SD* = 3.75) than TRF (*M* = 3.33, *SD* = 3.43), though this difference was not statistically significant, *t*(20) = −1.71, *p* = .10. In total, 16/21 participants (76%) vs. 20/23 participants (87%) reported at least one adverse event following the TRF and ADF protocols, respectively. However, this difference was not statistically significant (McNemar’s exact *p* = .687). In addition, three participants reported a total of 6 major life events, three during ADF and three during TRF, that were unrelated to the intervention itself. Importantly, these individuals were not identified as outliers for the primary outcome variables (a value + /- 3 standard deviations from the mean) and their inclusion did not alter the results, and therefore they were retained in all analyses.

**Table 3:** Adverse events by fasting protocol.

Type of Adverse Event	TRF	ADF
Body aches	19	24
Light-headedness	19	26
Nausea	9	13
Diarrhea	1	4
Vomiting	1	1
Headache	15	34
Other	6	6
**Total**	**70**	**108**

## Discussion

This study compared the acute psychological and behavioural effects of two IF protocols, ADF and TRF, on self-reported mood, hunger, diet satisfaction, and subjective sleep quality in young adult women. By examining these outcomes in a within-subject crossover design, our findings provide new insights into how these regimens are tolerated in the short term and highlight important differences in their psychological acceptability—factors that are central to adherence and long-term sustainability.

Contrary to the popular belief that fasting inevitably leads to irritability, mood changes observed here were protocol-specific and transient. Only ADF significantly disturbed mood, though this effect was limited to the first 24-hour fast. No mood changes were observed after the second 24-hour fast or after any 16-hour TRF fasts. These findings highlight the acute, transient nature of the mood changes following fasting, rather than a sustained effect. Interpretation of the findings should therefore be limited to the acute mood response to fasting, as opposed to any long-lasting fasting-related mood changes. Prior research has reported no mood effect from a single 24-hour fast [25], our results suggest instead that a 24-hour fast can temporarily disturb mood, but that this disruption diminishes rapidly, perhaps reflecting a process of emotional adaptation. Prior work has documented such adaptation over weeks of fasting, with novice fasters reporting more negative mood states than experienced fasters [46]. The present findings extend this evidence, demonstrating that adaptation may occur after just one 24-hour fast. However, the significant condition by time by order interaction for total mood disturbance suggests that mood responses to the fasting protocols may have been influenced by protocol order. Interestingly, mood disturbance was not associated with ketone or glucose levels on day 2 of ADF or TRF, suggesting that, under the conditions of this study, the psychological outcomes of fasting were not closely aligned with the measured physiological markers; however, this should not be interpreted as evidence that no relationship exists, as the non-significant findings may be a result of limited statistical power. Future research may be needed to clarify how acute psychological responses to fasting influence long-term physiological adaptations. Importantly, most participants in this study had no prior history of fasting, suggesting that this adaptation arose from the experience itself, rather than pre-existing fasting familiarity.

The absence of a mood change after the second 24-hour fast and across all 16-hour TRF fasts is notable when considered alongside the hunger results. Both protocols were associated with elevated hunger, consistent with prior evidence that a short-term caloric restriction is a metabolic stressor [14,17,18,47]. Yet, hunger did not consistently coincide with negative mood. This dissociation suggests that hunger alone is not sufficient to provoke a disturbance in mood. While some studies have found a direct correlation between hunger and mood [8], others point to psychological factors such as attentional focus or distraction (e.g., thinking about food or focusing on being hungry) as critical drivers of fasting-related mood disturbances [48]. Our findings support this latter interpretation, suggesting that once participants adapt to the fasting regimen (perhaps by learning how to divert attention away from the hunger cues), mood stability is maintained, even under elevated hunger. This challenges the common assumption that hunger directly produces irritability and highlights the importance of psychological processes in shaping fasting-related mood responses.

Participants consistently reported higher diet satisfaction during TRF compared to ADF, consistent with previous findings that TRF is perceived as more acceptable and easier to follow [19]. Importantly, this preference persisted even on ADF feeding days, when participants could eat freely, and mood and hunger states returned to baseline, whereas TRF restricted feeding to an 8-hour window. This suggests that diet satisfaction is shaped by factors beyond mood or hunger. Instead, elements such as predictability, structure, and compatibility with daily routines may play a central role, as indicated by prior survey data in real-world settings where willingness to adopt TRF not only depended on the feeding window, but also on lifestyle demands and individual motivations [49]. However, the significant interaction between intervention order and condition for diet satisfaction suggests that satisfaction with the protocol may have been influenced by protocol order and therefore, diet satisfaction findings should be interpreted cautiously. Although total fasting hours were equivalent across protocols, ADF was associated with more adverse events, suggesting it may be more physically taxing in the short term. In contrast to prior studies documenting relatively few adverse events with either protocol [50,51], in this study, the proportion of participants reporting at least one adverse event was relatively high (76% TRF and 87% ADF). This has important implications for real-world tolerability, as a higher number of reported adverse events may suggest lower tolerability, which, in turn, could lead to lower levels of adherence. However, diet satisfaction was measured using a single self-report question whose validity and sensitivity were not assessed. Future research should include a validated measure of diet satisfaction. Variations in study populations and intervention durations between our study and prior work suggest that tolerability may be context-dependent.

Neither ADF nor TRF significantly influenced subjective sleep quality, aligning with previous findings [24–28]. This null effect may reflect the resilience of our young, healthy sample to short-term metabolic stressors, the limited duration of the intervention, or the use of a single-item, self-reported measure of sleep quality. Other studies have observed fasting-related disruptions to aspects of sleep such as duration, sleep onset latency, or nighttime awakenings [29,30]. Thus, longer-term studies using both objective (e.g., actigraphy, polysomnography) and multidimensional subjective measures in more diverse populations are warranted to clarify whether IF affects sleep. This study has several strengths, including a crossover design that reduced individual variability, a within-subject comparison of two widely used fasting protocols, and the incorporation of both self-report and physiological adherence measures. Nonetheless, limitations should be noted. The sample was small, homogeneous, and composed exclusively of young, healthy women without a psychiatric diagnosis, limiting generalizability. Therefore, this research cannot be generalized to populations with clinically significant psychiatric symptoms and applies largely to young, healthy, fasting-naïve females. Additionally, no data on previous TRF and ADF experiences were collected, which could have influenced outcomes due to prior fasting exposure. The study was powered to detect moderate effects; smaller effects may have gone undetected. Furthermore, the repeated-measures ANOVA with pairwise deletion may be considered a limitation of incomplete repeated-measures data. Outcomes were assessed primarily through self-report, which may not capture subtle physiological changes. Moreover, protocol-related differences may have limited the interpretation of the effects of condition and time. Finally, the short duration precludes conclusions about long-term adherence, adaptation, and health outcomes.

In conclusion, this study highlights several novel insights into the acute psychological and behavioural effects of IF. First, mood disturbances following 24-hour fasting appear to be short-lived and resolve after only one prior exposure, suggesting rapid emotional adaptation. Second, hunger and mood responses were dissociated, challenging the assumption that hunger necessarily produces irritability. Third, participants reported greater satisfaction with TRF than with ADF, despite equivalent fasting hours, suggesting that TRF may be better tolerated than ADF during the initial days of implementation; however, longer interventions are required to determine whether this difference translates into greater adherence or long-term sustainability.

## Supporting information

S1 File Minimal Dataset. This file contains the minimal dataset required to reproduce the study results.
(XLSX)

S2 File Diet Satisfaction Questionnaire. This file contains the diet satisfaction questionnaire administered to participants. Responses were collected using a 5-point Likert scale.
(PDF)
