# Supplementary material for: Comparison of acute intermittent fasting interventions on mood, hunger, sleep quality, and diet satisfaction in females: A crossover experimental design
Source: PLoS One. 2026 Aug 26;21(8):e0356690. doi: 10.1371/journal.pone.0356690 (PMC13513999; doi:10.1371/journal.pone.0356690)
Supplement: Supplementary file 1 [file pone.0356690.s002.pdf]

The following question was developed by the study researchers for use in the current manuscript:

"If applicable, how would you rate your level of satisfaction with the diet so far? (Note: This pertains to whichever fasting protocol you are presently following)"

(1 = "I am not enjoying this diet at all" to 5 = "I am really enjoying this diet")

This question is available under the Creative Commons Attribution (CC BY 4.0) license.

© Amendola et al., 2025.
